# Improving the Efficacy of Deep-Learning Models for Heart Beat Detection on Heterogeneous Datasets

**DOI:** 10.3390/bioengineering8120193

**Published:** 2021-11-28

**Authors:** Andrea Bizzego, Giulio Gabrieli, Michelle Jin Yee Neoh, Gianluca Esposito

**Affiliations:** 1 Department of Psychology and Cognitive Science, University of Trento, 38068 Trento, Italy; andrea.bizzego@unitn.it; 2 Psychology Program, Nanyang Technological University, Singapore 639818, Singapore; giulio001@e.ntu.edu.sg (G.G.); michelle008@e.ntu.edu.sg (M.J.Y.N.); 3 Lee Kong Chian School of Medicine, Nanyang Technological University, Singapore 308232, Singapore

**Keywords:** ECG, deep neural networks, transfer learning

## Abstract

Deep learning (DL) has greatly contributed to bioelectric signal processing, in particular to extract physiological markers. However, the efficacy and applicability of the results proposed in the literature is often constrained to the population represented by the data used to train the models. In this study, we investigate the issues related to applying a DL model on heterogeneous datasets. In particular, by focusing on heart beat detection from electrocardiogram signals (ECG), we show that the performance of a model trained on data from healthy subjects decreases when applied to patients with cardiac conditions and to signals collected with different devices. We then evaluate the use of transfer learning (TL) to adapt the model to the different datasets. In particular, we show that the classification performance is improved, even with datasets with a small sample size. These results suggest that a greater effort should be made towards the generalizability of DL models applied on bioelectric signals, in particular, by retrieving more representative datasets.

## 1. Introduction

In medicine and other medical sciences, physiological recordings are widely employed to monitor and assess the health status of patients [1,2].

The possibility of using machine learning (ML) and artificial intelligence (AI) to automatize the extraction of physiological indicators from signals has been widely explored in recent years. Numerous studies have employed AI models on preprocessed physiological signals, for instance, to identify usable segments of pupillometry measures in infants [3], as well as ventricular hypertrophy [4], arrhythmia [5], muscle fatigue [6], and stress [7]. The employment of such techniques not only allows for a reduction in the amount of time and resources required for signal processing but also increases the reproducibility of the process while reducing the likelihood of human errors.

Among the available AI techniques, deep neural networks (DNNs) have proven to be one of the most promising [8,9]. As a family of machine learning methods, DNNs rely on the use of modular architectures based on multiple non-linear processing units (layers) to extract high-level patterns from data. Due to the hierarchical structure of the layers, DNNs progressively obtain high-level features from low-level representations [10], thus, transforming input data into a multi-dimensional representation that is used to solve the classification task [11].

The adoption of convolutional neural networks (CNNs) in applications based on medical data (bio images and physiological signals) is rapidly growing, with a wide range of applications [12,13,14,15,16]. Not exclusive to image processing, DNNs and CNNs have also been applied successfully to the analysis of physiological signals.

For instance, in a study by Wieclaw et al. [17], a DNN was successfully employed to design a biometric identification signal based on an electrocardiogram (ECG), while Mathews et al. [18] employed a DNN to identify ventricular and supraventricular ectopic beats. Moreover, Xu et al. [19] effectively employed a DNN to classify the type of heartbeat patterns (e.g., normal beat and arrhythmia) from raw ECG recordings. Similar procedures were employed on other physiological signals: for example, Yu and Sun [20] used a DNN to classify emotions from phasic and tonic components of the electrodermal activity (EDA), while Mukhopadhyay and Samui [11] employed DNN to classify limbic movements from electro-myogram (EMG) signals.

The identification of heart beats in ECG signals is one of the main tasks in clinical practice that uses physiological signals. Several methods and algorithms have been proposed [21,22,23,24]; however, regardless of its proven efficacy, the use of DNNs is still limited.

Silva and colleagues [25,26] presented an approach based on the combination of a CNN model with the Pan–Tompkins algorithm, a popular QRS complex detection algorithm. Despite the high effectiveness of the model proposed by Silva et al. [26], one of the main problems of a inaccurate identification of beats in the ECG signals may cause excessive false alarm problem, especially in Intensive Care Units (ICU) [27,28,29].

One of the greatest concerns of the application of DNN in clinical practices lies in the applicability of a model with signals for which it has received little or no training. For example, a model trained on a dataset consisting of signals recorded from healthy subjects may be employed in the ICU, where patients with arrhythmia may be present. Beat identification errors in medical settings cause false alarms that can make the identification of underlying conditions more difficult [30,31], and can affect both patient [32] and healthcare professionals wellbeing [33].

Multiple works have explored the possible application of DNN to different types of clinical diagnosis of cardiovascular diseases from the ECG signals. Promising methods have been proposed using different network architectures. For example, Sujadevi et al. [34] proposed a method based on a Gated Recurrent Unit that was able to achieve a 100% success rate in the identification of atrial fibrillation, while Al Rahhal et al. [35] employed convolutional neural networks (CNNs) for the diagnosis of ventricular ectopic and supraventricular ectopic beats.

A review conducted by Ebrahimi et al. [36] revealed that CNNs are among the most widely employed architecture for the study of ECG signals, being used in about 52% of the studies they investigated. More recently, Siontis et al. [37] confirmed the feasibility and potential value of the application of CNNs to the automated analysis of ECG signals, highlighting the successful results of such models on the diagnosis of hypertrophic cardiomyopathy [38], silent atrial fibrillation [39], and asymptomatic left ventricular dysfunction [40].

As such, before it can be extensively used in clinical practice, any AI-based solution has to be tested to prove its reliability. However, there are situations in which obtaining a dataset on which to train and test the model may be impossible, as obtaining training data from the population of interest may be difficult and expensive (e.g., rare conditions).

To overcome the problem of obtaining features that match those of interest, researchers have developed a technique, called transfer learning, that consists in the transferring of knowledge across domains [41] . A typical example of transfer learning consists in the training of a model on data that are similar but not exactly matching those of interest; this approach has been extensively used to transfer networks from general image classifications to clinical applications [42].

Farhadi et al. [43] , for example, successfully developed a model to recognize sign language trained on avatar signers and tested on a small dataset of human signers. Is it important to note that the two domains have to be similar, as the less similarity between the two sets of data, the lower the performances [44]. Applications of transfer learning on ECG signals have been tested as well.

Salem et al. [45] reported a study in which they tested the performances of a CNN on arrhythmia classification from images, with a model trained on generic images, while Van Steenkiste et al. [46] employed transfers learning to classify the ECG of horses using a network trained on human ECG recordings.

More recently, Weimann and Conrad [47] successfully tested the feasibility of employing transfer learning to classify periodic heart arrhythmia using a model trained on healthy subjects’ ECG recordings and small set of clinical recordings. Yet, little is known about the reliability of networks trained on healthy subjects in the classification on ECG signals recorded from individuals with different clinical conditions.

### Aim of This Study

The aim of this work is to verify the performance of using a convolutional deep neural network trained for a ECG beat detection task on the signals of healthy subjects, when signals recorded from individuals from a clinical population are fed to the model.

## 2. Materials and Methods

### 2.1. Datasets

The data used in this study were obtained from three datasets from Physionet [48] and from the WCS dataset [49].

Specifically, we used the MIT-BIH Normal Sinus Rhythm Database (https://doi.org/10.13026/C2NK5R, accessed on 23 November 2021) and the MIT-BIH Long-Term ECG database (https://doi.org/10.13026/C2KS3F, accessed on 23 November 2021), including long-term ECG recordings from 18 subjects and 7 subjects, respectively. The two datasets were merged to compose the NormalSinus+LongTerm subset. The MIT-BIH Arrhythmia Database [50] (https://doi.org/10.13026/C2F305, accessed on 23 November 2021), including 48 30-min ECG recordings from 47 subjects with clinical arrhythmias, was used to compose the Arrhythmia subset.

Finally, we used the WCS dataset [49] (https://doi.org/10.21979/N9/42BBFA, accessed on 23 November 2021), including ECG signals from 18 healthy subjects, simultaneously collected with a medical grade device (FlexComp acquisition unit, Thought Technology) and a wearable device (ComfTech HeartBand) in two experimental settings: baseline and movement, each lasting 5 min. In particular, we considered data collected during the baseline with the FlexComp device (Baseline FlexComp subset) and with the ComfTech device (Baseline ComfTech subset) to represent data collected with different medical grade and wearable devices during resting, i.e., where signals should show no contamination from artifacts due to body movement.

Then, we considered data collected during movement with the ComfTech device (Movement ComfTech subset) to represent data collected in real-life contexts, i.e., where signals are likely to be affected by movement artifacts.

Subsets were divided into two partitions, used for training (Training partition) and testing (Testing partition) the network models. Partitions were created by dividing the subjects in each dataset into two groups, with the Training partition including approximately 66% of the subjects, and the Testing partition including the remaining 33% of the subjects. The actual number of subjects composing each partition and subset is provided in Table 1.

### 2.2. Signal Processing

**Figure 1:**
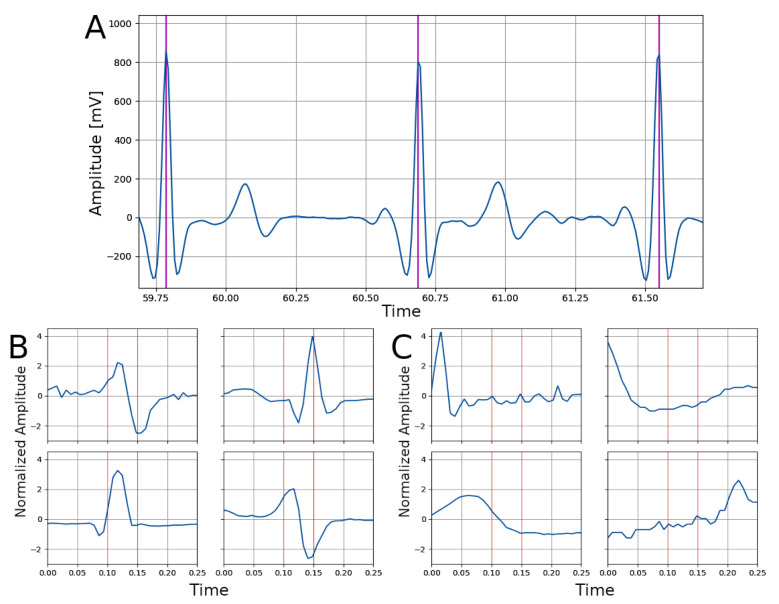
Signal processing steps on the ECG signals. (**A**) Portion of the original ECG data (from the Baseline ComfTech subset). Vertical lines indicate the position of the R peak indicating an heart-beat. (**B**) Examples of four samples belonging to the BEAT class: R peak between 0.1 to 0.15 s (indicated by the vertical red lines). (**C**) Examples of four samples belonging to the NO-BEAT class: R peak not present or not between 0.1 to 0.15 s (indicated by the vertical red lines).

All datasets used in this study included ECG signals and heart beat annotations, which were used to create and label ECG signal segments (see Figure 1A). Only the first 3600 s of long term recordings were considered. ECG signals were segmented using fixed-length non-overlapping portions (duration 0.25 s). Each segment was assigned to one of two classes: BEAT or NO-BEAT. The BEAT class was assigned if a heart beat was located between 0.1 and 0.15 s from the beginning of the segment; otherwise, the NO-BEAT was assigned. Examples of segments for each class are reported in Figure 1B,C.

Finally, the segment was resampled at 1000 Hz with linear interpolation to uniform the length of the segments to 250 samples. Our segmentation method is different from the one used in [25] and results in more unbalanced classes: in our subset, only ≈6–7% of the samples belonged to the BEAT class (in [25], it was 37.5%). For the processing of the signals, the pyphysio Python package was used [51]. No further preprocessing steps were applied on the signals in order to ensure that the performances of the classification network were not influenced by the preprocessing procedure.

### 2.3. Network Architecture

**Figure 2:**
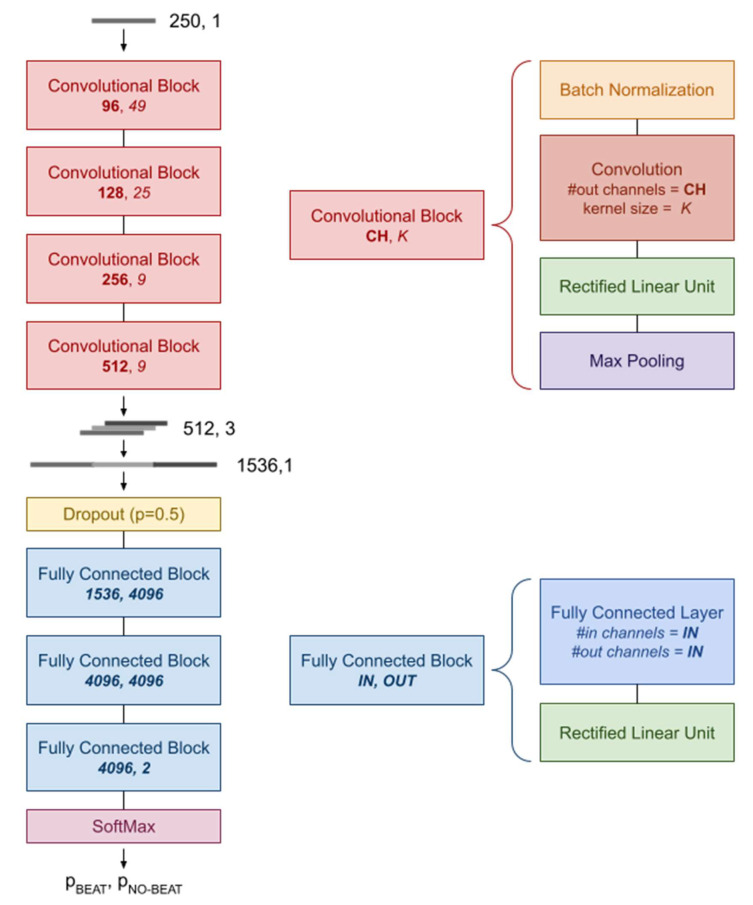
Schematic illustration of the network architecture used in this study.

The network architecture used in this study aims to replicate the one proposed by Silva and colleagues [25,26] and is composed of a Convolutional Part, followed by a Fully Connected Part. The Convolutional Part is composed of four convolutional blocks, each composed of four layers for one-dimensional data: (a) Batch Normalization [52], (b) Convolution (with variable number of output channels and kernel size), (c) Rectified Linear Unit activation [53], and (d) Max Pooling (with the kernel size set to 2). The four blocks differ in the number of output channels and kernel size of the Convolution layer (see Figure 2).

The Fully Connected Part (FCP) is composed by; (a) a Dropout layer (with the drop-out probability set to 0.5), (b) a sequence of three Fully Connected Blocks, each composed of a Fully Connected Layer and a Rectified Linear Unit activation [53], and (c) a SoftMax layer (see Figure 2).

### 2.4. Network Training and Transfer Learning

The training and testing of the network were performed on the Gekko Cluster of the High Performance Computing Centre (HPCC, Nanyang Technological University, Singapore). To train the network, only samples in the training partitions of the subsets were used. The training was iterated for 10 epochs, using all the segments, randomly divided into batches of 64 segments. The training was performed with back-propagation to minimize the Weighted Cross-Entropy Loss [54] between the true and predicted class.

Loss weights are set to 0.06 for the NO-BEAT class and to 0.94 for the BEAT class to compensate for the class imbalance. The AdaDelta algorithm [55] was used to optimize the network weights, with a learning rate of 0.01. The classification performance was evaluated using the Matthew Correlation Coefficient (MCC) [56] between true and predicted labels, on both the Training and Testing partitions. The bootstrap technique (100 repetitions on 25% of the samples randomly selected with replacement) was used to obtain the mean MCC with 90% confidence intervals.

The full network was only trained once, on the NormalSinus+LongTerm subset; for the other subsets, we adapted the trained network using the transfer learning method. Specifically, we loaded the weights resulting from the training on NormalSinus+LongTerm subset and re-trained only the weights of the Fully Connected Part while keeping the weights of the Convolutional Part.

### 2.5. Experiments

The aim of this study was to assess the performance and reproducibility of a beat detection neural network on signals from different populations and collected with different devices and in different contexts. We therefore designed three experiments, using different subsets and transfer learning:

(1) Experiment 1—The first experiment aimed at reproducing the results of Silva and colleagues [25]. In this experiment, we trained the network using samples in the Training partition of the NormalSinus+LongTerm subsets and evaluated the performance on both the Training and Testing partition of the same subset. The predictive performance was also assessed in terms of the percentage of positive predicted samples (*+p*, also known as Precision), sensitivity (*Se*, also known as Recall) and F-score (*F1*), to be able to compare the results with the Silva et al. study;
(2) Experiment 2—The second experiment aimed at evaluating the performance of the trained network on the Testing partition of the other subsets: (a) the Arrhythmia subset, representing a clinical population; (b) the Baseline FlexComp and (c) the Baseline ComfTech subsets, representing a normal population at rest with signals collected using another medical grade device and a wearable device respectively; (d) the Movement ComfTech subset, representing the same normal population during movement, using a wearable device;
(3) Experiment 3—The third experiment aimed at assessing the feasibility and impact of transfer learning the trained network on the same subsets. The trained network was retrained on the Training partitions and evaluated on the Training and Testing partitions.

## 3. Results

**Figure 3:**
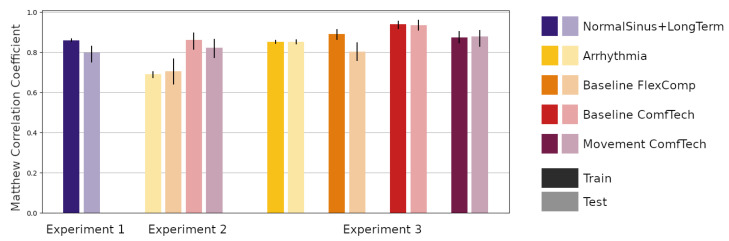
The Matthew Correlation Coefficient of the networks on different datasets and partitions. Vertical bars indicate 90% confidence intervals.

A summary of the predictive performances of the network in the three experiments, in terms of MCC with 90% CI of the prediction, are reported in Figure 3.

Regarding the first experiment (Experiment 1, NormalSinus+LongTerm subset), the network achieved an MCC = 0.860 (90% CI = [0.855, 0.866]) on the Training partition and MCC = 0.797 (90% CI = [0.751, 0.830]) on the Testing partition. In terms of the number of positive predicted samples, the percentage was *+p* = 86.7% (90% CI = [85.9, 87.6]%) on the Training partition and *+p* = 85.3% (90% CI = [81.3, 90.5]%) on the Testing partition, while the measured sensitivity of the network was *Se* = 87.3% (90% CI = [86.6, 88.2]%) and *Se* = 78.3% (90% CI = [71.9, 82.3]%) on the Training and Testing partitions, respectively. Finally, the F-score was *F1* = 0.870 (90% CI = [0.864, 0.876]%) on the Training subset and *F*1 = 0.815 (90% CI = [0.772, 0.853]%) on the Testing subset. A summary of the results in tabular form is reported in Table 2.

Moving forward to the second experiment (Experiment 2), the results, reported in Table 3, show that the performance of the network deteriorated when the model trained for Experiment 1 was applied to predict samples in the Arrhythmia (MCC = 0.690, 90% CI = [0.675, 0.703]) and Baseline FlexComp (MCC = 0.706, 90% CI = [0.642, 0.767]) subsets, while it obtained comparable results (although with larger CI) on the Baseline ComfTech (MCC = 0.861, 90% CI = [0.815, 0.895]) and Movement ComfTech (MCC = 0.822, 90% CI = [0.774, 0.865]) subsets.

Performing the transfer learning (Experiment 3) improved the performance of the network, although only the Fully Connected module of the network underwent a new training procedure. The performance of the network on the Training and Testing partitions of all the subsets were similar, with the exception of the performance on the Baseline FlexComp subset. A summary of the MCC for each partition and subset as well as the respective 90% CI are reported in Table 3.

## 4. Discussion

In this paper, we conducted three experiments to investigate the performance of a deep-learning model, trained on ECG signals collected from healthy subjects, on the identification of heart beats in signals collected from individuals from a clinical population or in signals collected with different devices.

Regarding the first experiment (Experiment 1), using our settings, the classification performance reported in Silva et al. [25] could not be achieved. While the experiment reported in this work and the methods employed in Silva et al. [25] are similar, there are substantial differences that could explain the discrepancies in the reported sensitivity and F-score of the two models. First, the networks employed in this study and in Silva et al. [25] present certain architectural differences, which could influence the overall results.

Additionally, in this work, no data augmentation procedure was performed, and the analyzed dataset included an unbalanced number of classes. Overall, while our network was able to identify heart beats with great accuracy, the differences between the current work and other networks highlight the importance of replication studies to effectively assess the feasibility and applicability of different neural network architectures for clinical tasks and the influence of diverse modules and procedures (e.g., Data Augmentation) on the performance of the networks.

In our second experiment (Experiment 2), we verified how a network trained on the signals recorded from healthy subjects (Experiment 1) performed when employed to identify beats in segments of signals recorded from subjects with clinical conditions or on segments of signals recorded with different devices. Our results represented two different situations.

Out of the four subsets of samples tested, a reduction in the performance of the network was observed in two cases (Arrhythmia and Baseline FlexComp), while similar or better performance was reported in the other two subsets (Baseline ComfTech and Movement ComfTech). One possibility for this behavior may be found in the nature of the devices used for the recording of ECG signals. Signals recorded with the ComfTech device may be less affected by noise, due to the lower sampling frequency and an internal preprocessing procedure conducted within such wearable devices.

Regarding the possibility of retraining the network on subsets of the datasets in order to explore the impact and feasibility of transfer learning (Experiment 3), our results indicated an increase in the performance of the network over the baseline performance (Experiment 1) for three out of the four datasets (Arrhythmia, Baseline ComfTech, and Movement Comftech), while a performance that was comparable to the baseline but superior to the one reported before transfer learning (Experiment 2) was reported for the Baseline Flexcomp dataset.

Overall, the findings reported for the third experiment confirmed the possible adoption of the network on segments collected from individuals with clinical conditions or on segments collected using different devices after a re-training procedure on a subset of the dataset was conducted.

Despite the good performance of our network, the drawbacks of the application of transfer learning to neurophysiologcal signals should be considered. Explainability plays a crucial role in the adoption of a deep learning network, where features are not estimated prior to the analysis—as in machine learning studies—but are automatically learned from the network itself. As such, despite the possible higher accuracy of the model, the interpretability of the results is more complicated, especially from the biological perspective [57,58,59,60].

Generally, when the explainability of the results plays a crucial role, employing deep neural networks alone is not a recommended practice. Concerning the applicability of the trained network to other domains (transfer learning), in order to successfully apply the model to a different problem, the network should be able to extract only generic patterns. The results of our second experiment, reported in Figure 3, show that the model performances was lower when employed to classify data collected from clinical situations or devices for which it had not been trained, suggesting that the model was learning more than generic patterns.

The computational effort required to adapt the network to a different kind of signal—the ECG of individuals with clinical conditions or ECG collected with a different device, in the current paper—was measured in Experiment 3. After being retrained with the addition of a portion of data from a new dataset, the performance of the network when employed to classify different signals improved and, in some cases, outperformed the original baseline (Experiment 1).

These results suggest that, when the model is trained on a multivariate dataset, its application with a transfer learning approach is more feasible. This is crucial especially for the possible application of deep neural networks in a clinical setting, where high accuracy of the models is required and for which the transparency and reproducibility of the results is essential [61].

While the clinical field may benefit from the adoption of machine learning models, whose possible, application for the medical and research fields has been explored with regard to different types of signals and clinical situations [3,49,62,63,64,65]. It is important to also consider the time and resources needed to train and deploy such models, as compared to the more simplistic heuristics of linear algorithms.

Future studies should consider testing the network proposed here on different datasets and reporting the performances both before and after transfer learning. Moreover, future works should compare the computational workload and performance of simpler heuristics of algorithms with more complex machine learning or neural network models.

## 5. Conclusions

In this paper, we presented three different experiments on the application of a deep-learning model to the identification of heart beats in ECG signals. Our results confirm the possibility of successfully employing a DNN to identify beats in ECG signals recorded from individuals with different medical backgrounds or collected with devices of a different nature (e.g., clinical or wearables), by adopting a transfer learning procedure that only retrains a section of the network on a subset of a different dataset.

## Figures and Tables

**Table 1:** Sample sizes of the subsets used in this study for each partition. N: number of subjects; Segments: number of segments; and %BEAT: percentages of segments in the BEAT class.

Dataset Name	Training			Testing		
	N	Segments	% BEAT	N	Segments	% BEAT
NormalSinus+LongTerm	17	240,000	7.37	8	80,000	8.47
Arrhythmia	32	230,000	6.19	16	110,000	7.07
Baseline FlexComp	12	14,748	6.48	6	7384	6.43
Baseline ComfTech	12	14,741	6.62	6	7385	6.19
Movement ComfTech	12	14,886	7.33	6	7443	6.68

**Table 2:** Performance of the Network on the Training and Testing partitions of the NormalSinus+LongTerm subset.

Metric	Training Partition	Testing Partition
MCC	0.860 [0.855, 0.866]	0.797 [0.751, 0.830]
*+p*	86.7% [85.9, 87.6]	85.3% [81.3, 90.5]
Sensitivity	87.3% [86.6, 88.2]	78.3% [71.9, 82.3]
F-score	0.870 [0.864, 0.876]	0.815 [0.772, 0.853]

**Table 3:** Performance (MCC and 90% CI) of the Network before (Experiment 2) and after (Experiment 3) retraining; for each subset and partition under investigation.

Dataset Name	Experiment 2	Experiment 3	
	Testing Partition	Training Partition	Testing Partition
Arrhythmia	0.690 [0.675, 0.703]	0.852 [0.844, 0.859]	0.852 [0.843, 0.861]
Baseline FlexComp	0.706 [0.642, 0.767]	0.852 [0.864, 0.913]	0.803 [0.760, 0.847]
Baseline ComfTech	0.861 [0.815, 0.895]	0.939 [0.917, 0.954]	0.935 [0.911, 0.960]
Movement ComfTech	0.822 [0.774, 0.865]	0.874 [0.846, 0.902]	0.879 [0.830, 0.907]

## Data Availability

Data used in this study are derived from public datasets. They can be retrieved usink the DOI links provided in the Datasets Section 2.1. Scripts to create the subsets, train the models and reproduce the results are publicly available at: https://gitlab.com/abp-san-public/dl-beat-detection (accessed on 23 November 2021).
